# Supplementary material for: Deglacial mobilization of pre-aged terrestrial carbon from degrading permafrost
Source: Nat Commun. 2018 Sep 10;9:3666. doi: 10.1038/s41467-018-06080-w (PMC6131488; doi:10.1038/s41467-018-06080-w)
Supplement: Supplementary file 1 — Supplementary Information [file 41467_2018_6080_MOESM1_ESM.pdf]

Supplementary Information for

Deglacial mobilization of pre-aged terrestrial carbon from thawing permafrost

Winterfeld et al.

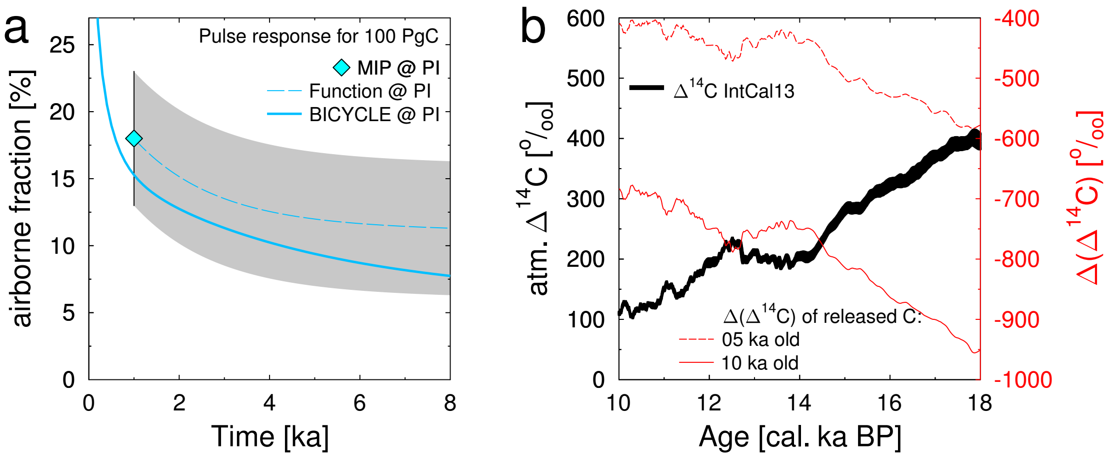

Supplementary Figure 1. Additional information on the carbon cycle simulations. (a) Comparing the model response (airborne fraction) of the global carbon cycle model BICYCLE with other models for a pulse of 100 PgC injected to the atmosphere for pre-industrial (PI) climate conditions. MIP: results of a model intercomparison project[64] restricted to 1 kyr, but extrapolated here by results of a regression analysis of an Earth system model[65]. Uncertainty band of 2σ is obtained from MIP for 1 kyr and its relative width is used to show the likely uncertainty range or model spread for longer time periods (grey area). (b) Prescribed difference in ^{14}C of the released carbon to the atmosphere ($\Delta(\Delta^{14}C)$) as function of time and pre-depositional age (5 or 10 kyrs). IntCal13 atmospheric $\Delta^{14}C$ for comparison.

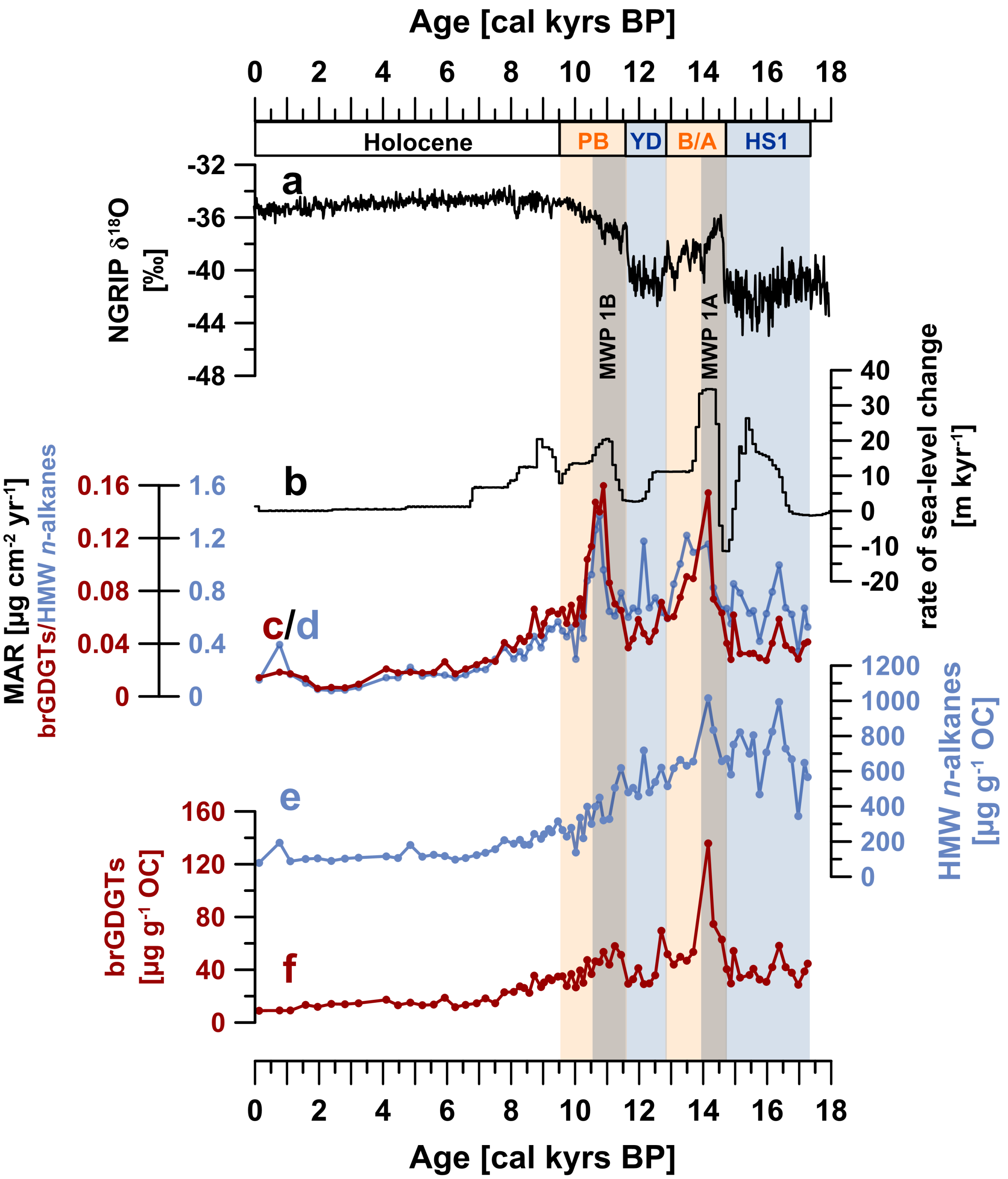

Supplementary Figure 2. Additional biomarker data of core SO178-136. (a) Greenland NGRIP $\delta^{18}O$[68]; (b) rate of global sea-level change[14]; mass accumulation rate (MAR) of (c) branched glycerol dialkyl glycerol tetraethers (brGDGTs) and (d) high molecular weight (HMW) *n*-alkanes (C_{27}, C_{29}, C_{31} and C_{33}); (e) concentration of HMW *n*-alkanes and (f) brGDGTs.

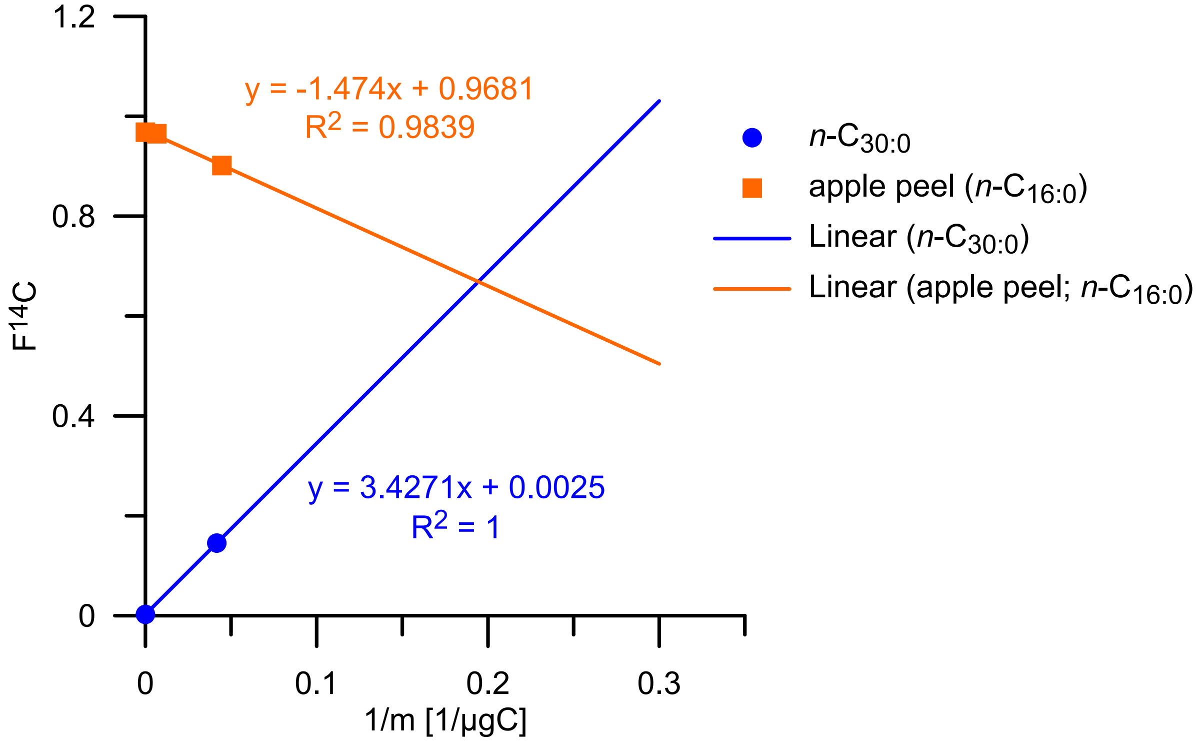

Supplementary Figure 3. Graphical assessment of the $F^{14}C$ value and mass of the process blank ($F^{14}C_{blank}$ & m_{blank}) based on the AMS ^{14}C results of modern and fossil standard material shown in Table S2. The intercept of the two regression lines yielded a $F^{14}C_{blank}$ of 0.6776 and a m_{blank} of 5.08 µg carbon. The uncertainty for $F^{14}C_{blank}$ ($\sigma F^{14}C_{blank}$) and m_{blank} (σm_{blank}) were determined by the regression coefficient R^2 of the *n*-$C_{16:0}$ data set. Because there were only two *n*-$C_{30:0}$ samples, R^2 of this data set was ignored. Based on the R^2 of 0.9839, the $\sigma F^{14}C_{blank}$ would be 0.0109. According to ref. 57 (see Methods), the R^2-value of 0.9839 implies that both, the $F^{14}C_{blank}$ and the m_{blank}, are predicted with 98.39% certainty by the respective regression line. Therefore, these authors inferred that σm_{blank} can be determined by: $\sigma m_{blank} = m_{blank}*(1-R^2)$. Based on $R^2 = 0.9839$ the equation yields $\sigma m_{blank} = 0.08$ µg C. It has to be acknowledged that σm_{blank} and $\sigma F^{14}C_{blank}$ are likely underestimated since the uncertainties of the *n*-$C_{30:0}$-dataset could not be taken into account.

Supplementary Table 1. Non-corrected, raw data of AMS analyses of long-chain *n*-alkanoic acids expressed as $F^{14}C$ of the cores SO178-13-6 and LV28-4-4 and their respective samples sizes given in µg carbon. $\sigma F^{14}C$ represents the measurement uncertainty from the AMS for each sample.

Sample depth (cm below surface)	Deposition age (mid-point) [cal kyrs BP]	*n*-alkanoic acid	$F^{14}C \pm \sigma F^{14}C$	Sample size [µg C]	ETH ID number
Core SO178-13-6					
55-65	0.76	*n*-C26:0	0.6252 ± 0.0058	108	59360.1.1
695-705	5.92	*n*-C26:0	0.3112 ± 0.0037	132	59359.1.1
1435-1445	10.02	*n*-C26:0[a]	0.1637 ± 0.0033	211	59355.1.1
1435-1445	10.02	*n*-C28:0[a]	0.1598 ± 0.0032	200	59357.1.1
1805-1815	11.97	*n*-C26:0	0.1813 ± 0.0043	223	59354.1.1
2033-2041	14.16	*n*-C26:0[a]	0.1280 ± 0.0027	170	59352.1.1
2335-2342	17.26	*n*-C26:0	0.0865 ± 0.0025	151	59351.1.1
Core LV28-4-4					
54-56	0.71	*n*-C26:0	0.5843 ± 0.0069	42	49686.1.1
54-56	0.71	*n*-C28:0	0.5694 ± 0.0080	33	49687.1.1
751-753	8.29	*n*-C28:0	0.1782 ± 0.0035	107	49688.1.1
860-862	11.78	*n*-C26:0	0.1303 ± 0.0029	99	49690.1.1
860-862	11.78	*n*-C28:0	0.1428 ± 0.0028	118	49691.1.2
926-928	16.0	*n*-C26:0	0.1286 ± 0.0029	85	49695.1.1

[a]66% split of sample

Supplementary Table 2. AMS ^{14}C results of modern and fossil standard material used for blank determination expressed as $F^{14}C$ and their respective samples sizes given in µg carbon. $\sigma F^{14}C$ represents the uncertainty from the AMS measurement. The mean $F^{14}C$ of the apple peel bulk was used for the blank assessment.

Standard material	Sample size [µg C]	$F^{14}C \pm \sigma F^{14}C$	ETH ID number
Unprocessed samples			
Sigma-Aldrich *n*-C30:0 unprocessed		0.0025 ± 0.0007[a]	-
Apple peel bulk	100	1.0311 ± 0.0038	64615.1.1
Apple peel bulk	249	1.0263 ± 0.0026	70188.1.1
Apple peel bulk	258	1.0279 ± 0.0026	70122.1.1
Apple peel bulk mean	-	***1.0284 ± 0.0030***	-
Processed samples			
Apple peel *n*-C16:0	151	0.9650 ± 0.0078	59306.1.1
Apple peel *n*-C16:0	22.4	0.9013 ± 0.0083	59307.1.1
Sigma-Aldrich *n*-C30:0	24	0.1453 ± 0.0038	59361.1.1
Calculated "methylation" value of unprocessed samples[b]			
Apple peel bulk		0.9681	
Sigma-Aldrich *n*-C30:0		0.0025	

[a]from ref. 58

[b]for ^{14}C blank assessment and corrections see Methods for more details
